# Discovering Low-Dimensional Descriptions of Multineuronal Dependencies

**DOI:** 10.3390/e25071026

**Published:** 2023-07-06

**Authors:** Lazaros Mitskopoulos, Arno Onken

**Affiliations:** School of Informatics, University of Edinburgh, Edinburgh EH8 9AB, UK; aonken@inf.ed.ac.uk

**Keywords:** copula, weighted NMF, non-parametric vine copula, neural dependence structures

## Abstract

Coordinated activity in neural populations is crucial for information processing. Shedding light on the multivariate dependencies that shape multineuronal responses is important to understand neural codes. However, existing approaches based on pairwise linear correlations are inadequate at capturing complicated interaction patterns and miss features that shape aspects of the population function. Copula-based approaches address these shortcomings by extracting the dependence structures in the joint probability distribution of population responses. In this study, we aimed to dissect neural dependencies with a C-Vine copula approach coupled with normalizing flows for estimating copula densities. While this approach allows for more flexibility compared to fitting parametric copulas, drawing insights on the significance of these dependencies from large sets of copula densities is challenging. To alleviate this challenge, we used a weighted non-negative matrix factorization procedure to leverage shared latent features in neural population dependencies. We validated the method on simulated data and applied it on copulas we extracted from recordings of neurons in the mouse visual cortex as well as in the macaque motor cortex. Our findings reveal that neural dependencies occupy low-dimensional subspaces, but distinct modules are synergistically combined to give rise to diverse interaction patterns that may serve the population function.

## 1. Introduction

Information processing in the brain relies on the coordinated activity of neuronal circuits on multiple spatial and temporal scales. The neural population function is dynamically shaped by a rich mixture of distributed and interdependent processes ranging from representations of sensory inputs to signals related to behavioral outputs or internal states. While past research focused mainly on characterizing neural codes by looking at properties of single neurons, recent years have seen a shift towards a population-level approach [1,2,3]. This shift was largely enabled by significant advances in imaging technologies [4] and tools for high-yield electrophysiology [5] which allowed for simultaneous recordings of large populations of neurons. These complex high-dimensional neural datasets provide a unique opportunity to examine the structure of population codes, but substantial challenges still exist from a data analytic standpoint. While encoding properties of single neurons have been studied extensively [6,7,8], making sense of collective population activity can be considerably more challenging. Population responses can exhibit complex multiscale spatiotemporal dynamics and non-trivial low- and higher-order interactions among neurons [9,10,11,12,13]. Moreover, these features can potentially be modulated by multiple variables related to the brain state or behavior such that it becomes difficult to determine how particular multineuronal responses are associated with certain stimuli or experimental conditions. Thus, in order to make sense of population activity, it is necessary to understand whether and how emergent population functions such as multivariate dependencies within neural ensembles and with behavioral variables are important for information processing over and above what can be performed by single neurons.

Existing literature on neural dependencies has predominantly focused on pairwise shared response variability between neurons, measured as linear correlations where the noise is assumed to follow Gaussian statistics [14,15,16,17,18]. Typically, a distinction is made between signal or stimulus correlations and noise correlations. The former term refers to tuning similarities between neurons with respect to particular stimuli and the latter term refers to shared neural variability beyond what can be accounted for by the stimuli and would be attributed to functional connectivity [18]. While such a linear approach has undoubtedly been useful as an approximation of neural dependencies, there are some concerns that suggest it might be yielding an impoverished or even misleading picture. First of all, spike trains consist of discrete spike counts that are characterized by positively skewed distributions, especially when examined in fine temporal scales, as a typical recording session of a neuron will mostly contain none or one spike within a given time bin. Higher spike counts are progressively less likely. These properties make spike trains unsuitable for modeling with Gaussian distributions, which are continuous and symmetric [19]. Moreover, neural dependencies quite often deviate significantly from the elliptical shapes that are characteristic of Gaussian joint variability models and instead tend to be heavy tailed [20]. Finally, a number of studies have discovered that cortical neurons can interact in groups larger than just pairs, although whether these high-order correlations limit or enhance the information content in neural codes remains contested [10,11,12,13,21,22]. Therefore, dissecting the structure of multivariate dependencies in neural populations is an important goal for neuroscience and one that requires an alternative approach that can address the aforementioned challenges.

A suitable alternative approach for studying multivariate dependencies is using copulas, a statistical tool that has been widely used in economics [23], but has received comparatively much less attention in neuroscience [19,24,25,26,27,28]. Copulas can be thought of as a description of the structure that underlies how individual random variables are coupled and produce the joint observations at hand. Unlike Pearson correlations which yield a single value, copulas are probability distributions with dimension equal to the number of entangled random variables under examination and thus can provide a much more detailed account of neural dependencies [29]. However, there are a few considerations when applying copulas on neural data. One challenge is that the copula theory has been built for continuous variables, whereas data in neuroscience research are often discrete (e.g., spike counts) or contain interactions between discrete and continuous variables with vastly different statistics (e.g., spikes with local field potentials or behavioral measurements such as running speed or pupil dilation). This challenge can be overcome with the use of additional probabilistic tools such as the distributional transform [30] or continuous convolutions [31], which transform discrete data into pseudo-continuous data such that one can still obtain consistent copulas for discrete and mixed settings. Another consideration with copula-based approaches is whether to use parametric or non-parametric descriptions of copula densities for characterizing dependence structures. Parametric families of copulas assume a certain dependence shape and are a powerful tool for inference, but they might also impose rigid assumptions that may result in mischaracterizing the actual dependence structure. Moreover, multivariate parametric copulas are quite limited in number and assume a particular type of dependence structure for all variables that can ignore potentially rich and heterogeneous interaction patterns. A common alternative for multivariate copula modeling is to decompose dependencies into a cascade of unconditional and conditional bivariate copulas organized into hierarchical tree structures called vines or pair copula constructions [32]. This formulation allows for a joint model that can flexibly incorporate various dependence structures. A number of previous studies have employed vine copulas in mixed variable settings with parametric models [28,33,34,35,36], while another line of research has explored various non-parametric alternatives that evade the need for making any assumptions regarding dependence structures [37,38,39,40].

In our previous work [41], we followed a fully non-parametric approach for capturing single-neuron margins and copula densities by using Neural Spline Flows (NSF) [42], which is a class of normalizing flows [43]. We found that NSFs performed similarly to existing non-parametric estimators while allowing for faster and more flexible sampling. Additionally, using the NSF framework made it possible to uncover neural dependence structures that would have been challenging to capture with common parametric copulas. However, this flexibility of non-parametric methods comes at the cost of sacrificing ease of summarization for these dependence structures. This is especially the case with recordings of larger neural populations since the number of copulas in vine formulations grows quadratically with population size. While it may seem like non-parametric copula-based approaches result in prohibitively large numbers of objects to work with, the space of dependence structures described by vine copula densities is usually sparse, as the majority of pairs in the vine tree are independent [20]. Furthermore, it is plausible to suggest that even within the subspace of non-independent pairs, dependence structures are likely to share some features such as asymmetric tail dependence. Following this rationale, our aim in this study was to uncover such shared low dimensional features which would be easier to visualize and draw insights from. Finding such features requires a dimensionality reduction technique such as non-negative matrix factorization (NMF), which is suitable for non-negative input data and yields an interpretable parts-based representation [44]. Although NMF is usually a good choice for feature discovery problems, application on copula densities with heavy tail dependencies that can potentially overlap with more light-tailed ones can be challenging. NMF representations can be distorted when datasets have overly dominant features [45]. A potential solution to this issue is to introduce a weight matrix that has a normalizing function. A few studies have explored adding weights to NMF specifically by multiplying the observation vectors with their inverse probabilities to adjust the importance NMF places on them for reconstruction [46,47]. Taking into account the approaches in the aforementioned studies, we decided to construct weight matrices for every estimated bivariate copula density by utilizing the inverse cumulative probabilities of single neuron spike count histograms. We thus followed a weighted non-negative matrix factorization framework (WNMF), which we validated on synthetic data against NMF and subsequently applied to neural recordings. Our findings shed light on the complexity of multineuronal interactions and point to fascinating new avenues for research into neural dependencies.

## 2. Materials and Methods

### 2.1. Copulas

**Figure 1:**
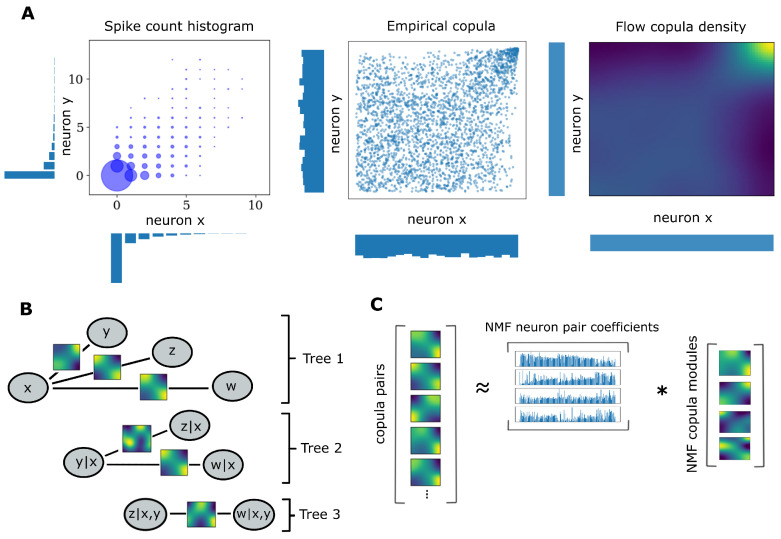
Mixed vine copula flows and NMF decomposition. (**A**) Spike train samples from two neurons can be decomposed into their margins and a copula. Empirical copulas are extracted by transforming the samples to uniform through the distributional transform. (**B**) Graphical illustration of a C-vine for 4 variables. Nodes and edges of the first tree denote the variables and bivariate dependencies, respectively. Edges of subsequent trees denote dependencies that condition on one or more variables. (**C**) Decomposition of pair copulas into non-negative coefficients for neuron pairs and copula factors.

Multivariate interactions in neural populations can be described probabilistically by means of copulas. A full characterization of the multivariate statistics of population spiking responses would require modeling the joint probability distribution for these responses. However, one can tackle instead an alternative formulation of that problem as according to Sklar’s theorem [48], every multivariate cumulative distribution function (CDF) $F_{x}$ can be decomposed into its margins, in this case the single-neuron distributions for spike counts $F_{1},\ldots F_{d}$, and a copula *C* (Figure 1A) such that:

(1) $$F_{x}\left(x_{1},\ldots ,x_{d}\right)=C\left(F_{1}\left(x_{1}\right),\ldots ,F_{d}\left(x_{d}\right)\right)$$

Copulas are multivariate CDFs with support on the unit hypercube and uniform margins and their shape encapsulates the dependence structure between random variables. Such representations of dependence are not bound by assumptions such as linearity or gaussianity as is the case with Pearson’s correlation coefficient [29]. Following Sklar’s theorem, it is possible to obtain copulas from joint distributions using:

(2) $$C\left(u_{1},\ldots ,u_{d}\right)=F_{x}\left(F_{1}^{-1}\left(u_{1}\right),\ldots ,F_{d}^{-1}\left(u_{d}\right)\right),$$

Equivalently, one can also construct proper joint distributions by entangling margins with copulas. Going from sample space to copula space and the opposite is possible via the probability transform *F* and its generalized inverse, the quantile transform $F^{-1}$. The probability transform maps samples to the unit interval: $F\left(X\right)\to U∼U_{\left[0,1\right]}$, where $U_{\left[0,1\right]}$ denotes the uniform distribution on the unit interval. Two key properties of copulas are that they are unique and invariant under increasing margin transformations when data come from continuous distributions. However, these properties do not hold true for discrete distributions [30], and additional tools are required for consistent mapping to copula space. One such tool is the distributional transform:

(3) $$G\left(X,V\right)=F_{x-}\left(x\right)+V\left(F_{x}\left(x\right)-F_{x-}\left(x\right)\right)$$

where $F_{x-}\left(x\right)=Pr\left(X<x\right)$ as opposed to the regular expression for the CDF, $F_{x}\left(x\right)=Pr\left(X<=x\right)$, and *V* is a random variable uniformly distributed on [0, 1] independent of *X*. This transformation extends the probability transform by adding uniform jitters in between discontinuous intervals in the support of discrete variables. Discrete samples that are subjected to the distributional transform result in quasi-continuous empirical copulas, the densities of which can be estimated with tools designed for continuous data. In practice, when one is interested in dependencies among joint observations, it is more convenient to work with copula densities instead of cumulative distributions. Estimating the copula densities amounts to characterizing dependence structures in joint observations. An example pair of neurons is illustrated in Figure 1A. Spiking distributions with non-negligible tails that are transformed to copula space tend to feature probability mass concentration in the corners. The empirical copula in this example displays asymmetric heavy tail dependence on the upper-right corner and the copula density can be estimated with non-parametric methods as outlined later in the text.

### 2.2. Pair Copula Constructions

Copula-based approaches can suffer from the curse of dimensionality with an increasing number of interacting variables. Pair copula constructions [49] can tackle this challenge by factorizing multivariate dependencies into a cascade of bivariate conditional and unconditional copulas. Given that the space of possible factorizations is prohibitively large, most applications use vine copula structures, a special type of pair copula construction [32]. Vines are hierarchical sets of trees that account for a specific graph of multivariate interactions among elements of the distributions and assume conditional independence for the rest. A C-Vine (Figure 1B) is one in which each tree in the hierarchy has a node that serves as a connection hub to all other nodes. This property makes the C-Vine an attractive structure for modeling multivariate neural dependencies since functional connectivity patterns in neural recordings tend to feature a few neurons that interact with many other neurons [28]. The C-Vine decomposes the joint distribution *f* into a product of its margins and conditional copulas *c*.

(4) $$f_{X}\left(x_{1},\ldots ,x_{d}\right)=\prod_{k=1}^{d}f\left(x_{k}\right)\prod_{j=1}^{d-1}\prod_{i=1}^{d-j}c_{j,i+j|1,\ldots ,j-1}\left(F\left(x_{j}|x_{1},\ldots ,x_{j-1}\right),F\left(x_{i+j}|x_{1},\ldots ,x_{j-1}\right)\right)$$

where $c_{i,j|A}$ denotes the pair copula between elements *i* and *j* given the elements in the set *A*, which is empty in the first tree, but increases in the number of elements with deeper trees.

### 2.3. Copula Flows

As in our previous work [41], we modeled the margin and copula densities non-parametrically using Rational-Quadratic Neural Spline Flows (NSF) [42], a specific type of normalizing flow [43]. These flows are a class of generative models that can construct arbitrary probability densities using smooth and invertible transformations to and from simple probability distributions. In essence, this is an application of the change of variables formula:

(5) $$p_{x}\left(x\right)=p_{u}\left(T^{-1}\left(x\right)\right)\det\left|\frac{\partial T^{-1}\left(x\right)}{\partial x}\right|,$$

where $p_{x}\left(x\right)$ is the density of the observations and $p_{u}$ is the base density of random variable $U=T^{-1}\left(X\right)$, which is a known and convenient distribution such as the normal or the uniform distribution. The transformation *T* is usually a composition of invertible transformations as in an artificial neural network with a certain number of layers and hidden units that are optimized during training. The determinant of the Jacobian matrix for *T* keeps track of volume changes. The main advantage of normalizing flows lies in the fact that they enable harnessing the flexibility of artificial neural networks for probability density modeling, while invertibility allows for simple and efficient sampling from the base distribution.

As previously [41], we chose the uniform distribution on [0, 1] as a base distribution for NSF so that backward and forward flow transformations for the margins approximate the probability/distributional and the quantile transform, respectively. The reason for choosing NSF [42] for modeling both margin and copula densities was that they combine the flexibility of non-affine flows while maintaining easy invertibility by approximating a quasi-inverse with piecewise spline-based transformations around knot points of the mapping.

### 2.4. Sequential Estimation and Model Selection

The procedure we followed for fitting the C-vine model with NSF to data was the same as in our previous work, where it is described in more detail [41]. Briefly, we decided the order of the variables for the C-vine model based on a heuristic, namely, the sum of absolute value Kendall’s taus that we calculated for each neuron in relation to every other neuron. Then, we fit each margin with NSF and then proceeded with bivariate copulas of the surface layer of the tree. For each subsequent layer, we estimated conditional margins via h-functions [50]:

(6) $$h\left(x,y\right)=F\left(x|y\right)=\frac{\partial C(x,y)}{\partial y},$$

whereby h(x,y) is the function that allows obtaining the conditional marginal distribution F(x|y) by taking the partial derivative of the copula linking variables *x* and *y* with respect to *y*. Then, conditional copulas can be obtained by transforming those conditional margins according to Equation (2). We followed the simplifying assumption [51] for the conditional copulas and conducted a random search procedure [52] to determine the optimal number of hidden layers, hidden units and number of spline knots for the NSF. We held out a validation set containing 10% of the data to evaluate performance for different NSF configurations. Lastly, since a large part of the vine was expected to contain independent pairs of variables [20], we followed a heuristic by which we tested every empirical copula for independence using a two-dimensional Kolmogorov–Smirnov test [53]. In essence, this test was comparing copulas with samples from a two-dimensional uniform distribution and deemed them independent if the two did not exhibit statistically significant differences for p<0.05. This heuristic reduced the computational load of fitting NSF models.

### 2.5. Weighted Non-Negative Matrix Factorization

NMF [44] is an algorithm that learns a parts-based representation of a data matrix *X*. In our study, this matrix consists of the densities for all bivariate copulas (Figure 1C left). More precisely, we discretized and vectorized the two-dimensional densities of the bivariate copulas and stacked the resulting vectors. This data matrix is factorized into a product of two other matrices, *H* and *W*, corresponding to the copula modules (Figure 1C right) and the neuron pair coefficients (Figure 1C middle). All three matrices must be non-negative, a constraint which leads to a sparse, parts-based representation of *X* because modules can only act in a cumulative way. When using NMF, the goal is to find such a representation that approximates *X* with *W* and *H* having lower rank than *X*, thus conducting dimensionality reduction. The rationale behind such a low-dimensional description is to distill the multivariate neural dependencies into a set of *H* modules that capture interaction patterns among neurons that are shared across the population and a set of coefficients *W* that define the degree to which each neuron pair is composed of one or more of these modules. The most common method to learn *W* and *H* such that we minimize $||X-{WH||}^{2}$, i.e., the Frobenius norm of the difference between the real and the reconstructed data, is by initializing *W* and *H* with random non-negative numbers and updating them until they become stable. Iterative updates are implemented according the following multiplicative update rules [44]:

(7) $$W_{i+1}\leftarrow W_{i}\cdot \frac{XH_{i}^{T}}{W_{i}H_{i}H_{i}^{T}}$$

(8) $$H_{i+1}\leftarrow H_{i}\cdot \frac{W_{i}^{T}X}{W_{i}^{T}W_{i}H_{i}}$$

where subscript *i* denotes the iteration of the optimization procedure. In our study, we used a modified version of NMF, which we refer to throughout the text as weighted non-negative matrix factorization (WNMF). In this framework, the input matrix was weighted in order to aid detection of overlapping dependence structures with considerable differences in scale. We constructed weight matrices *V* according to:

(9) $$Vx,y=\left(F_{x}^{-1}\left(x\right)-U\right)⊗\left(F_{y}^{-1}\left(y\right)-U\right)$$

where for every neuron pair (x,y) we took the outer product of their inverse CDFs $F_{x}^{-1}\left(x\right)$, subtracted from the uniform CDF *U*. In order for the weight values to transition smoothly between high importance (tails) and low importance areas, we convolved the weight matrices with a Gaussian window of standard deviation equal to 5 bins in density space. Moreover, we added rotated versions of the outer product matrices such that all 4 corners of the copula space would be weighted heavily. Our approach and aim were therefore different from other examples of weighted NMF procedures we encountered in the literature [46,47]. Instead of weighting observation vectors according to the frequency they appear in, we aimed to differentially scale each copula density such that erroneous reconstruction in the tail regions is penalized more compared to other regions. We adjusted the NMF multiplicative update rules by adding *V* as follows:

(10) $$W_{i+1}\leftarrow W_{i}\cdot \frac{VXH_{i}^{T}}{VW_{i}H_{i}H_{i}^{T}+\alpha_{1}\cdot \left|W\right|+0.5\cdot \alpha_{2}\cdot {\left||W|\right|}^{2}}$$

(11) $$H_{i+1}\leftarrow H_{i}\cdot \frac{W_{i}^{T}VX}{W_{i}^{T}VW_{i}H_{i}+\alpha_{1}\cdot \left|H\right|+0.5\cdot \alpha_{2}\cdot {\left||H|\right|}^{2}},$$

where we also added regularization terms for both L1 and L2 regularization, the strengths of which were determined by the parameters $\alpha_{1}$ for L1 and $\alpha_{2}$ for L2. The values for these two parameters were tuned using 5-fold speckled cross-validation [54]. This cross-validation procedure is useful for optimal rank selection in dimensionality reduction analyses as it holds out a random set of entries for each fold, which it treats as missing values that can be used to calculate validation errors.

### 2.6. Synthetic Data

We validated our WNMF framework for describing dependence structures on a set of synthetic data consisting of two-dimensional copula densities on a 100 by 100 points grid. The densities were estimated using NSF on empirical copulas from 20,000 pairs of dependent Poisson samples generated using the mixed vines package developed by Onken and Panzeri [28]. The densities exhibited dependence structures that were characterized by known parametric copula families, namely the Clayton copula (theta = 5) and the Frank copula (theta = 6) [55]. The former displays asymmetric heavy tail dependence, i.e., the concentration of probability mass in one of the corners whereas in the latter, mass is symmetrically allocated along the correlation path. We designed 3 different test cases with data matrices that contained 2, 4 or 6 different copula densities that were Clayton, Frank or rotated versions of them. Each different density was present in 20 rows of each matrix.

### 2.7. Experimental Data

We evaluated our framework on two different datasets involving recordings of neural activity. The first one consisted of 2-photon calcium imaging recordings in the mouse primary visual cortex (V1), that were collected at the Rochefort lab (see [56] for more details). Briefly, V1 layer 2/3 neurons labeled with the calcium indicator GCamP6s were imaged while the animal was headfixed, freely running on a cylindrical treadmill and navigating through a virtual reality environment (160 cm). Mice were trained to lick at a specific location (120–140 cm) along the virtual corridor in order to receive a reward. Over the course of 5 days, mice learned to lick within the reward zone and V1 responses were modulated in conjunction with learning. Our analysis was based on the spike trains that were deconvolved from the calcium transients (see [56] for more details). We limited our scope of investigation to population activity (102 neurons) of mouse ST260 on day 4 of the experiment when the animal was an expert at the task.

The second dataset in our analysis was a session from electrode array recordings of primary motor cortex (M1) responses of head restrained macaque monkeys during a delayed reaching task [57]. We focused on the spiking activity of 81 neurons within a 900 ms window (10 ms bins) after a go cue was given for initiating reach movements towards a target position. There were 8 different target positions arranged in a circle. We included only those trials during the baseline epoch where movement was successful in reaching the cued target position.

## 3. Results

We propose WNMF to identify structures in C-vine models composed of non-parametric copulas and estimated with normalizing flows. Since dependencies in a vine tree are known to be sparse and display common features in studies using parametric methods, we aimed to discover shared structures in copula-based models by decomposing copula densities into lower dimensional copula modules and neuron pair coefficients. We assessed this approach on synthetic data generated with parametric copulas models, and on recordings from a mouse visual cortex and macaque motor cortex.

### 3.1. Validation on Synthetic Data

**Figure 2:**
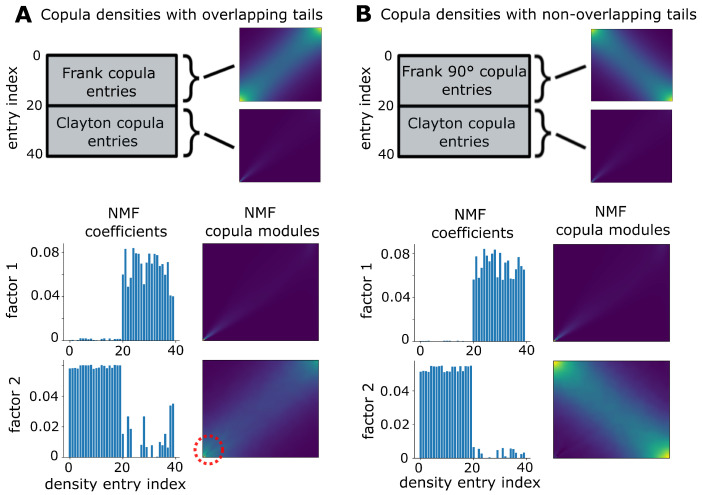
Overlapping tail dependencies are challenging for standard NMF. (**A**) A matrix of 20 Frank copula flow densities and 20 Clayton copula flow densities (Top illustration) is reduced to 2 NMF factors with coefficients (blue bar plots) and copula modules. Bottom left tail dependence structures overlap, which does not affect the Clayton copula factor but leads to incorrect identification for the Frank copula left tail, which is highlighted with a red dashed circle. This is in contrast to (**B**), where the rotated (90°) Frank copula tail regions do not overlap with those of the Clayton copulas (top illustration), allowing for correct detection of both copula types.

The addition of a weighting matrix to NMF was motivated by preliminary findings we had on synthetic data. One of these “toy” datasets we generated was composed of copula density estimates, namely 20 examples of Frank copulas and another 20 examples of Clayton copulas. We estimated the copula densities with NSF as outlined in the methods section out of copula samples generated using the mixed vines package [28]. Then, we applied standard NMF with two factors. The method was able to correctly identify the Clayton density as one of the factors and the coefficients with high values were corresponding to the actual entries in the data with that copula (Figure 2A, NMF factor 1). However, the other factor that corresponded to Frank copulas did not faithfully capture the bottom left tail region (Figure 2A, NMF factor 2 highlighted with red dashed circle). This discrepancy was not present when the data contained Frank copulas rotated by 90° instead of non-rotated ones. Presumably, since Frank and Clayton tail regions were not overlapping in this example, NMF identified the Frank copula density correctly (Figure 2B, factor 2).

While NMF can usually separate overlapping structures due to its non-negativity constraint, outstanding features can dominate and potentially contaminate other factors in a low-dimensional representation [45,57]. When the outstanding features are highly active neurons, one usually normalizes population firing rates to level the field. In the case of copula densities, however, the main source of the issue lies in tail regions where one may encounter massive scale differences in probability concentration among different cases. Therefore, our approach needed to be more targeted, i.e., place more emphasis on the representation of the tails. The weight matrices we constructed individually for each bivariate copula were designed to accommodate for this need.

**Figure 3:**
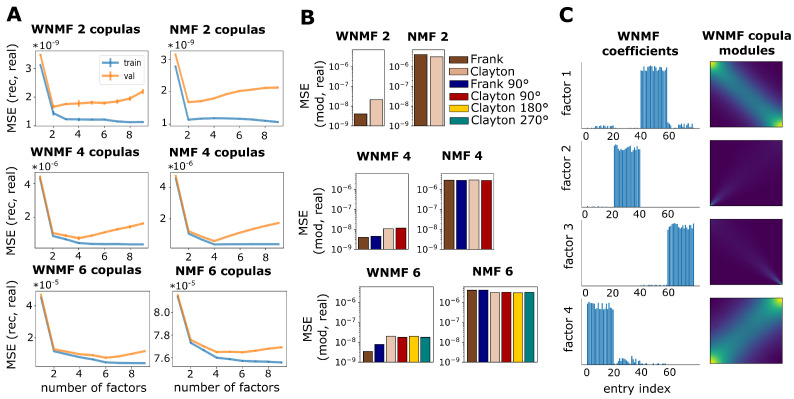
WNMF outperforms standard NMF in identifying dependence structures with overlapping tails. (**A**) Train (blue) and validation (orange) MSE over 5 folds for 1 to 8 factors. Error is computed for the real data matrix against the reconstructed one for WNMF (left column of line plots) and for NMF (right column of line plots). Both methods were tested on data containing either 2, 4 or 6 different copulas. (**B**) Bar plots depict MSE of the real copula densities versus the copula modules identified by WNMF (left column of bar plots) and NMF (right column of bar plots). Y axes are in logarithmic scale. Bar colors correspond to different copula families, namely Frank (brown) and Clayton (beige), as well as their rotated versions used in the cases with 4 and 6 copulas, namely Frank 90° (navy blue), Clayton 90° (red), Clayton 180° (yellow) and Clayton 270° (teal). (**C**) Illustration of WNMF factorization for the case of 4 copulas. Bar plots depict WNMF coefficients and density plots depict the copula modules discovered by WNMF.

We sought to validate whether weighting improved NMF performance by comparing WNMF to standard NMF on synthetic data that were generated in the same way as described above. There were three different cases with two, four and six copulas contained in the data matrices, each represented by 20 entries, which resulted in matrices with 40, 80 and 120 entries, respectively. Speckled cross validation for reconstruction error relative to the original matrices yielded similar curves for WNMF and NMF for two and four copulas and the validation error minimum landed at the correct number of factors (Figure 3A top and middle panels). The same was not true for the case with six different copulas, where the optimal number of factors was not clearly indicated by the NMF validation error curve. On the contrary, the WNMF validation error reached a clearer minimum at six, which was the correct one, and had overall lower train and validation errors (Figure 3A bottom panel). A different picture emerged from examining the error between the copula modules from NMF and WNMF and the real copula densities. Under this lens, WNMF had a clear advantage over NMF as MSE error was much lower for all cases (note the scale difference on the vertical axes of bar plots in Figure 3B). In all three cases, the lowest error we observed was for WNMF copula modules corresponding to Frank copulas (Figure 3B bar plots in left column). Almost the opposite trend seemed to occur for NMF in the data containing two and six copulas, where the error regarding Frank copula modules was slightly higher compared to that of Clayton copula modules (Figure 3B bar plots in right column). These results suggest that while both NMF and WNMF can identify low-dimensional structures such that adequate overall reconstruction of data is possible, WNMF is advantageous in terms of faithful discovery of important features of copula densities, as it can circumvent scale differences in probability density concentration. The reason why this is not obvious in the reconstruction error is because linear combinations of discovered features can still achieve good reconstruction quality despite these features being misidentified. An illustration of the copula modules along with neatly grouped entry membership coefficients that WNMF discovered in the example with four different copulas is provided in Figure 3C.

### 3.2. WNMF Identifies Shared Latent Structures of Neural Dependencies in Visual Cortex

In our previous work [41], we studied neural dependencies with NSF-based C-vine copulas in a small subset of mouse V1 layer 2/3 neurons that were recorded during a virtual navigation task [56]. Even in this small subset we identified features such as asymmetric tail dependencies that would have been ignored by conventional pairwise correlations analyses. In the present study, we sought to explore the range of multivariate dependencies encountered in the entire recorded population’s deconvolved spiking responses with the same NSF-based C-vine copula framework. Since this approach produces a large number of bivariate unconditional and conditional copulas, applying WNMF aids both visualization and interpretation of neural dependencies.

**Figure 4:**
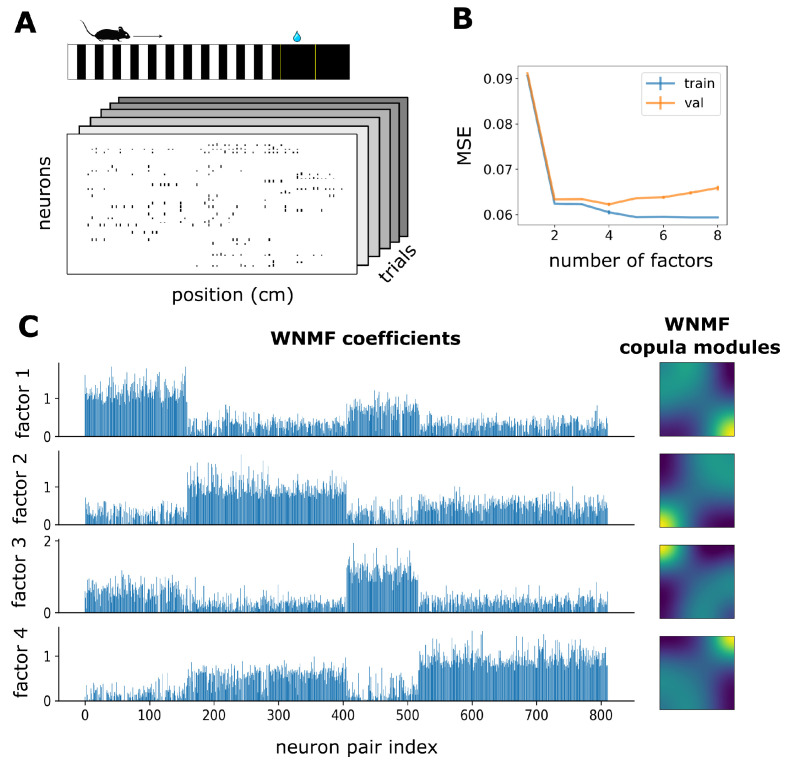
WNMF discovers structured and synergistic copula modules in mouse V1 responses. (**A**) Illustration of mouse navigating a virtual environment with grating stimuli until a designated reward zone (at 120–140 cm), where it is required to lick in order to receive a water reward. Neurons were recorded across a number of trials on each day of the experiment and their activity was binned with respect to the position of the mouse in the virtual corridor. (**B**) Average across 5 folds train (blue) and validation (orange) MSE for WNMF across different numbers of factors. (**C**) WNMF 4-dimensional representation of visual cortex copula dependence structures. Blue Bar plots depict WNMF coefficients specific to each neuron pair. Density plots depict copula modules discovered by WNMF.

We focused on the recording session on day 4 for mouse ST260 [56], where the animal was well-acquainted with the requirements of the task, which involved running through a virtual reality corridor and licking to receive a reward (Figure 4A top illustration). The data consisted of deconvolved spiking activity from calcium recordings of 102 neurons over 204 trials, binned with respect to position (bin size was 20 cm) (Figure 4A bottom illustration). We estimated single neuron spike count marginal distributions and the cascade of bivariate copula densities by means of NSF in a sequential fashion for each tree across the vine. Out of the 5151 copulas, 4340 were independence copulas according to two-dimensional Kolmogorov–Smirnov tests (p>0.05). We found that even the remaining 811 bivariate copulas were in fact occupying a much lower dimensional subspace, as four WNMF factors achieved an adequate approximation of the dependence structures therein (Figure 4B). The copula modules discovered by WNMF were arranged in a way that covered all four corners of copula space (Figure 4C WNMF copula modules). These modules have uniform margins and thus are proper copulas as well. In order to uncover potential groupings of neuron pairs we sorted the coefficients with respect to the module which they are most strongly associated with. By doing so we found that these coefficients were distributed in a way that implied it is a synergistic combination of these modules that gives rise to a diverse set of dependence structures from simple parts (Figure 4C WNMF coefficients). For example, a sizable part of the neuron pairs are strongly associated with the fourth copula module, which indicates a positive codependence that is only occurring after a certain regime of neural activity. However, this pattern seems to almost never occur in isolation as the 2nd copula module is also a part that shapes dependence structures for this particular group of neuron pairs and to a varying degree for each pair. Such an arrangement suggests a flexible and graded membership of neural dependencies into groups exhibiting different degrees of non-linear and non-Gaussian behavior. It might also allow for behavior that is close to linear and Gaussian, with more suppressed tail dependencies.

**Figure 5:**
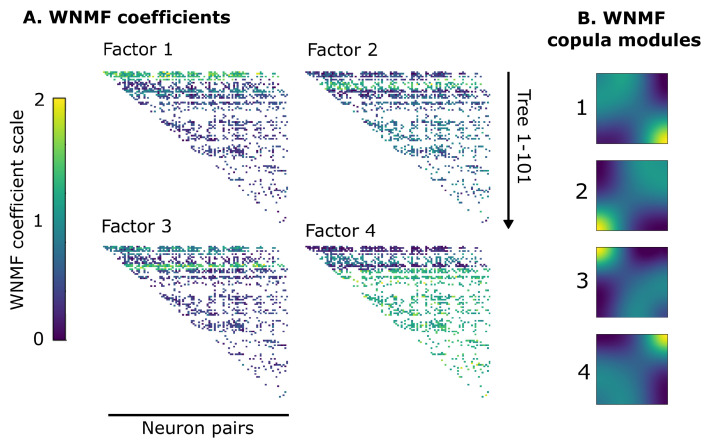
WNMF factors are distinctly grouped across different trees. (**A**) WNMF coefficients from the same decomposition as Figure 4. The 4 factors have been plotted in a 2 by 2 arrangement where each block consists of rows of color-coded values of WNMF for a particular tree, starting from tree 1 at the top until tree 101 at the bottom. Warmer and colder colors illustrate the spatial divides across superficial and deeper trees with respect to copula modules per neuron pairs. Blank spaces denote independent neuron pairs. (**B**) Same WNMF copula modules as in Figure 4 depicted again here for illustration purposes.

Of additional interest was the fact that the WNMF copula modules exhibited a rather sharp divide between the first few trees and the deeper ones. The former were predominantly associated with the first copula module (Figure 5) followed by the second and third modules playing a bigger role in subsequent trees, while the the fourth copula module played a more decisive role in describing neural interactions in deeper trees, potentially implying the presence of cliques of neurons with probably similar patterns of interaction that are being revealed by the pair copula construction.

### 3.3. WNMF Identifies Shared Latent Structures of Neural Dependencies in Macaque Motor Cortex

**Figure 6:**
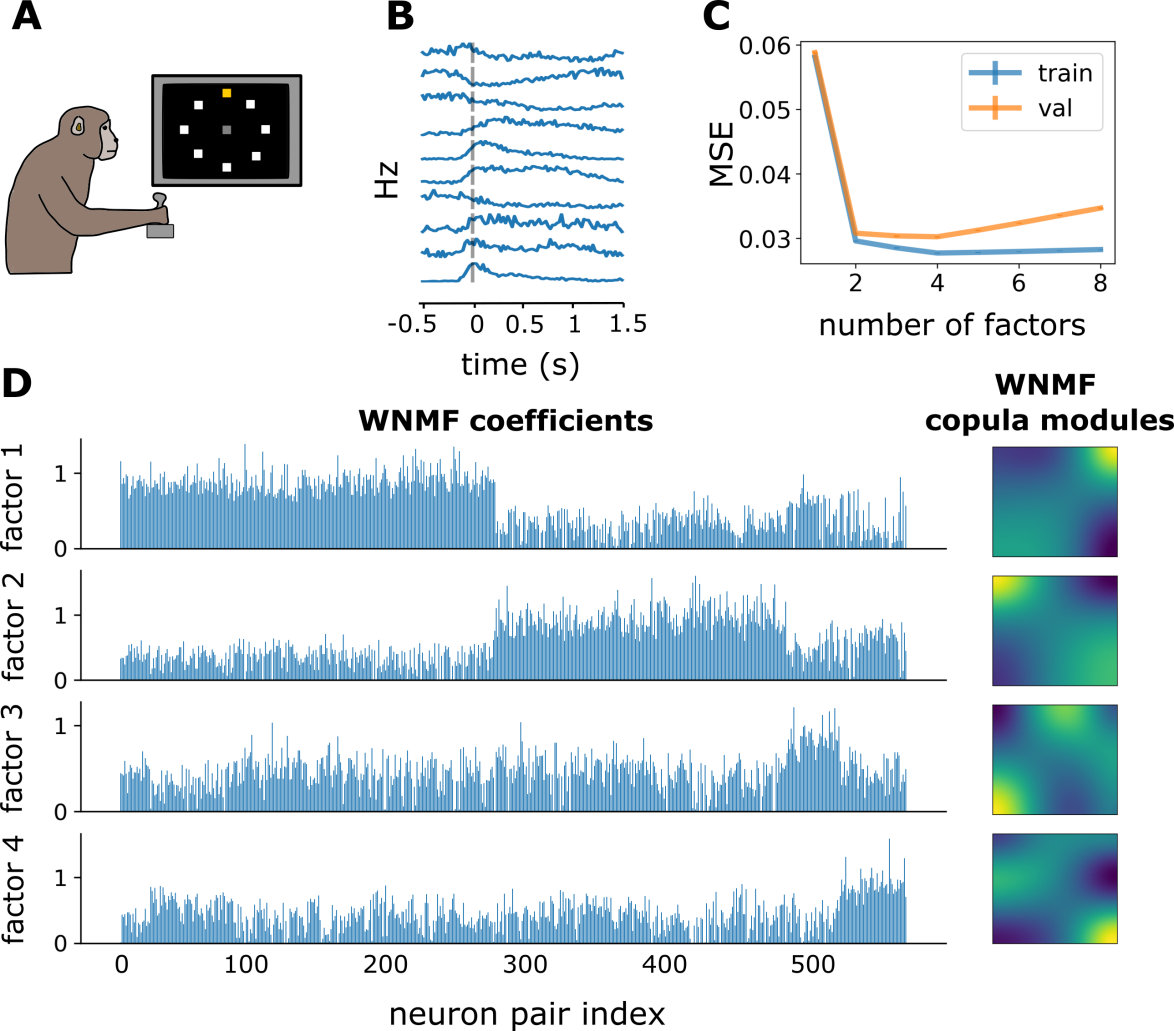
WNMF discovers main and distributed copula modules in macaque motor cortex. (**A**) Illustration of a macaque monkey moving a lever as part of a delayed center-out reaching task [57]. Monitor has 8 different targets drawn as white squares, while the cued target is highlighted with yellow. (**B**) Average firing rate (Hz) across trials of different target presentations. Horizontal axis indicate the time interval we chose for analysis, i.e., −0.5 to 1.5 s with respect to the go cue for movement initiation. (**C**) Average across 5 folds train (blue) and validation (orange) MSE for WNMF across different numbers of factors. (**D**) WNMF 4-dimensional representation of motor cortex copula dependence structures. Blue bar plots depict WNMF coefficients specific to each neuron pair. Density plots depict copula modules discovered by WNMF.

Subsequently, we evaluated our framework on electrode array recordings from the macaque primary motor cortex during a delayed center-out reaching task [57]. The experiment required that upon the start of every trial the monkey would look at a screen showing eight possible targets arranged in a circular formation (Figure 6A). Out of these eight targets, a different one was cued at every trial and after a varying time interval a go cue was given to the monkey. On that cue, the monkey was supposed to move a lever to initiate movement towards the target that had been previously cued. Movements that correctly reached the cued target were rewarded. For our analysis, we focused on one recording session during the baseline epoch of the experiment and included only successful trials. This part of the session contained neural spiking activity of 81 motor cortical neurons, which we aligned with respect to the go cue, and specifically during a time interval extending from 0.5 s before to 1.5 s after the go cue (Figure 6B). Similarly to the analysis on visual neurons, we estimated single neuron spike count marginal distributions and the cascade of bivariate copula densities by means of NSF in a sequential fashion for each tree across the vine. Out of the 3240 copulas, 2664 were independence copulas according to two-dimensional Kolmogorov–Smirnov tests (p>0.05). The remaining 576 bivariate copulas were similarly found to occupy a lower dimensional space, which could be approximated with four WNMF factors (Figure 6C).

In a somewhat similar fashion to what we observed in the case of the primary visual neurons, these copula modules also spanned the entirety of copula space and occupied all four corners (Figure 6D WNMF copula modules). However, there were also shapes in this example as with module 3 and 4, the mass of which extended in areas adjacent to the diagonal correlation path. These two modules are good examples of dependence structures that would be challenging for parametric copulas to capture. After sorting the coefficients with respect to the module they are more strongly associated with, groupings were revealed mostly for modules 1 and 2, while modules 3 and 4 seemed to have a distributed presence across the entirety of non-independent neuron pairs.

## 4. Discussion

In this study, we conducted an analysis that focused on uncovering and summarizing complex dependence structures in neural population responses. We built up on our previous work [41] where we introduced a C-vine copula-based approach coupled with normalizing flows that allows for flexible non-parametric modeling of neural dependencies. Here, we extended that framework to explore the range of multivariate dependence structures that underlie population function. As this approach yields a large number of copulas, we proposed to leverage the fact that vine copula representations are sparse and low dimensional [20] and apply WNMF on the copula densities to aid visualization and interpretation of our findings. Using synthetic data, we found that WNMF outperforms NMF in distinguishing copula densities with overlapping features. Moreover, as few as 4 WNMF factors were enough for an adequate representation of the latent dependence structures both in a mouse visual cortex as well as in a macaque motor cortex.

To properly contextualize our findings, it is necessary to note that our framework has several components that are worth discussing separately to appreciate where our approach deviates from other ones in the literature. Non-parametric modeling of dependence structures allows for more flexibility compared to using parametric families of copulas, especially when faced with arbitrary interaction patterns. While one can fit parametric mixture models to increase their flexibility, these models can also inflate the number of components. Doing so can potentially overestimate the complexity of the dependence structure. It would most likely be a challenge for parametric models or mixtures of them to fit copulas like the ones in modules 3 and 4 in Figure 6C. Moreover, the graded membership of copula modules in different groups where probability densities at the tails are present to different degrees (Figure 4C) would have been either difficult to capture or would require additional mixture components. Therefore, non-parametric modeling with normalizing flows is advantageous regarding ease of fitting and very low levels of bias towards a certain expected dependence structure.

A downside of non-parametric modeling is the large number of copulas they yield that are not easy to make sense of, especially with larger ensembles of neurons. However, our findings demonstrated that there is only a low fraction of non-independent copulas in the recordings we analyzed and the rest do not need to be considered to study neural dependencies. In our C-vine fits, most conditional elements were independent, suggesting that the C-vine decomposition is appropriate for the data at hand. Besides the sparseness though, we also found that the number of factors required to describe the set of non-independent copulas was surprisingly low, albeit still allowing for a fair amount of variation in dependencies as linear combinations of simple modules (Figure 4C).

These findings raise interesting questions regarding the function of neural dependencies in multineuronal responses. These questions have so far been discussed mostly in the context of linear correlations, which is rather limiting considering the complexity observed in these responses. Whether the synergistic combinations we found in copula modules in the mouse visual cortex (Figure 4C) have a functional role or aid information transmission to downstream neurons would be something worth exploring in future research. Similarly, it would be interesting to ask whether primary motor neurons during a reaching task like the one at hand might predominantly take part in positive (Figure 6D module 1) or negative codependence modes (Figure 6D module 2) while other modules may be part of dependence structures that are maintained in the background. The scope of our analysis does not make it possible to assess whether such modules of multineuronal dependencies have an information enhancing or limiting role. Moreover, since we were largely interested in validating WNMF and discovering population-wide low-dimensional features in multivariate dependence structures, we did not include behavioral variables in our analysis. However, complex dependencies with these variables are quite likely to exist and can be the subject of future research directions with non-parametric copula-based approaches. An interesting question for future research to explore is to which degree decoding behavioral variables from neural activity might be affected positively or negatively by the presence and shape of complicated neural dependencies, and whether describing them with WNMF offers insights into distinctive features of information enhancing or limiting structures which is not accessible otherwise. Moreover, particular behavioral patterns, e.g., a mouse running as opposed to being stationary might be associated with different dependence structures manifested in different copula modules, or changes in the dimensionality of the space of dependence structures. Such potential research directions make a compelling case for a more detailed exploration of the structure of neural dependencies.

## Data Availability

The neural datasets we used in our study are not publicly available but can become available upon request from the authors of the original publications [56,57].

## References

[1] Saxena S., Cunningham J.P. (2019). Towards the neural population doctrine. Curr. Opin. Neurobiol..

[2] Vyas S., Golub M.D., Sussillo D., Shenoy K.V. (2020). Computation through neural population dynamics. Annu. Rev. Neurosci..

[3] Urai A.E., Doiron B., Leifer A.M., Churchland A.K. (2022). Large-scale neural recordings call for new insights to link brain and behavior. Nat. Neurosci..

[4] Chen X., Leischner U., Varga Z., Jia H., Deca D., Rochefort N.L., Konnerth A. (2012). LOTOS-based two-photon calcium imaging of dendritic spines in vivo. Nat. Protoc..

[5] Jun J.J., Steinmetz N.A., Siegle J.H., Denman D.J., Bauza M., Barbarits B., Lee A.K., Anastassiou C.A., Andrei A., Aydın Ç. (2017). Fully integrated silicon probes for high-density recording of neural activity. Nature.

[6] Wu M.C.K., David S.V., Gallant J.L. (2006). Complete functional characterization of sensory neurons by system identification. Annu. Rev. Neurosci..

[7] Rolls E.T., Treves A. (2011). The neuronal encoding of information in the brain. Prog. Neurobiol..

[8] Kass R.E., Amari S.I., Arai K., Brown E.N., Diekman C.O., Diesmann M., Doiron B., Eden U.T., Fairhall A.L., Fiddyment G.M. (2018). Computational neuroscience: Mathematical and statistical perspectives. Annu. Rev. Stat. Appl..

[9] Hurwitz C., Kudryashova N., Onken A., Hennig M.H. (2021). Building population models for large-scale neural recordings: Opportunities and pitfalls. Curr. Opin. Neurobiol..

[10] Ohiorhenuan I.E., Mechler F., Purpura K.P., Schmid A.M., Hu Q., Victor J.D. (2010). Sparse coding and high-order correlations in fine-scale cortical networks. Nature.

[11] Yu S., Yang H., Nakahara H., Santos G.S., Nikolić D., Plenz D. (2011). Higher-order interactions characterized in cortical activity. J. Neurosci..

[12] Shimazaki H., Amari S.I., Brown E.N., Grün S. (2012). State-space analysis of time-varying higher-order spike correlation for multiple neural spike train data. PLoS Comput. Biol..

[13] Montangie L., Montani F. (2017). Higher-order correlations in common input shapes the output spiking activity of a neural population. Phys. A Stat. Mech. Appl..

[14] Zohary E., Shadlen M.N., Newsome W.T. (1994). Correlated neuronal discharge rate and its implications for psychophysical performance. Nature.

[15] Brown E.N., Kass R.E., Mitra P.P. (2004). Multiple neural spike train data analysis: State-of-the-art and future challenges. Nat. Neurosci..

[16] Moreno-Bote R., Beck J., Kanitscheider I., Pitkow X., Latham P., Pouget A. (2014). Information-limiting correlations. Nat. Neurosci..

[17] Kohn A., Coen-Cagli R., Kanitscheider I., Pouget A. (2016). Correlations and neuronal population information. Annu. Rev. Neurosci..

[18] Panzeri S., Moroni M., Safaai H., Harvey C.D. (2022). The structures and functions of correlations in neural population codes. Nat. Rev. Neurosci..

[19] Onken A., Grünewälder S., Munk M.H., Obermayer K. (2009). Analyzing short-term noise dependencies of spike-counts in macaque prefrontal cortex using copulas and the flashlight transformation. PLoS Comput. Biol..

[20] Kudryashova N., Amvrosiadis T., Dupuy N., Rochefort N., Onken A. (2022). Parametric Copula-GP model for analyzing multidimensional neuronal and behavioral relationships. PLoS Comput. Biol..

[21] Pillow J.W., Shlens J., Paninski L., Sher A., Litke A.M., Chichilnisky E., Simoncelli E.P. (2008). Spatio-temporal correlations and visual signalling in a complete neuronal population. Nature.

[22] Michel M.M., Jacobs R.A. (2006). The costs of ignoring high-order correlations in populations of model neurons. Neural Comput..

[23] Jaworski P., Durante F., Härdle W.K. (2012). Copulae in Mathematical and Quantitative Finance: Proceedings of the Workshop Held in Cracow, 10–11 July 2012.

[24] Jenison R.L., Reale R.A. (2004). The shape of neural dependence. Neural Comput..

[25] Berkes P., Wood F., Pillow J. (2008). Characterizing neural dependencies with copula models. Adv. Neural Inf. Process. Syst..

[26] Onken A., Grünewälder S., Munk M., Obermayer K. (2008). Modeling short-term noise dependence of spike counts in macaque prefrontal cortex. Adv. Neural Inf. Process. Syst..

[27] Sacerdote L., Tamborrino M., Zucca C. (2012). Detecting dependencies between spike trains of pairs of neurons through copulas. Brain Res..

[28] Onken A., Panzeri S. (2016). Mixed vine copulas as joint models of spike counts and local field potentials. Adv. Neural Inf. Process. Syst..

[29] Faugeras O.P. (2017). Inference for copula modeling of discrete data: A cautionary tale and some facts. Depend. Model..

[30] Genest C., Nešlehová J. (2007). A primer on copulas for count data. ASTIN Bull. J. IAA.

[31] Nagler T. (2018). A generic approach to nonparametric function estimation with mixed data. Stat. Probab. Lett..

[32] Aas K., Czado C., Frigessi A., Bakken H. (2009). Pair-copula constructions of multiple dependence. Insur. Math. Econ..

[33] Song P.X.K., Li M., Yuan Y. (2009). Joint regression analysis of correlated data using Gaussian copulas. Biometrics.

[34] de Leon A.R., Wu B. (2011). Copula-based regression models for a bivariate mixed discrete and continuous outcome. Stat. Med..

[35] Smith M.S., Khaled M.A. (2012). Estimation of copula models with discrete margins via Bayesian data augmentation. J. Am. Stat. Assoc..

[36] Panagiotelis A., Czado C., Joe H. (2012). Pair copula constructions for multivariate discrete data. J. Am. Stat. Assoc..

[37] Racine J.S. (2015). Mixed data kernel copulas. Empir. Econ..

[38] Geenens G., Charpentier A., Paindaveine D. (2017). Probit transformation for nonparametric kernel estimation of the copula density. Bernoulli.

[39] Schallhorn N., Kraus D., Nagler T., Czado C. (2017). D-vine quantile regression with discrete variables. arXiv.

[40] Nagler T., Schellhase C., Czado C. (2017). Nonparametric estimation of simplified vine copula models: Comparison of methods. Depend. Model..

[41] Mitskopoulos L., Amvrosiadis T., Onken A. (2022). Mixed vine copula flows for flexible modelling of neural dependencies. Front. Neurosci..

[42] Durkan C., Bekasov A., Murray I., Papamakarios G. Neural spline flows. Proceedings of the 33rd Conference on Neural Information Processing Systems (NeurIPS 2019).

[43] Rezende D., Mohamed S. Variational inference with normalizing flows. Proceedings of the International Conference on Machine Learning.

[44] Lee D.D., Seung H.S. (1999). Learning the parts of objects by non-negative matrix factorization. Nature.

[45] Russo A.A., Bittner S.R., Perkins S.M., Seely J.S., London B.M., Lara A.H., Miri A., Marshall N.J., Kohn A., Jessell T.M. (2018). Motor cortex embeds muscle-like commands in an untangled population response. Neuron.

[46] Guillamet D., Vitria J., Schiele B. (2003). Introducing a weighted non-negative matrix factorization for image classification. Pattern Recognit. Lett..

[47] Zhou Q., Feng Z., Benetos E. (2019). Adaptive noise reduction for sound event detection using subband-weighted NMF. Sensors.

[48] Sklar M. (1959). Fonctions de repartition an dimensions et leurs marges. Publ. Inst. Stat. Univ. Paris.

[49] Bedford T., Cooke R.M. (2002). Vines—A new graphical model for dependent random variables. Ann. Stat..

[50] Czado C. (2019). Analyzing Dependent Data with Vine Copulas.

[51] Haff I.H., Aas K., Frigessi A. (2010). On the simplified pair-copula construction—Simply useful or too simplistic?. J. Multivar. Anal..

[52] Bergstra J., Bengio Y. (2012). Random search for hyper-parameter optimization. J. Mach. Learn. Res..

[53] Fasano G., Franceschini A. (1987). A multidimensional version of the Kolmogorov—Smirnov test. Mon. Not. R. Astron. Soc..

[54] Owen A.B., Perry P.O. (2009). Bi-cross-validation of the SVD and the nonnegative matrix factorization. Ann. Appl. Stat..

[55] Nelsen R.B. (2007). An Introduction to Copulas.

[56] Henschke J.U., Dylda E., Katsanevaki D., Dupuy N., Currie S.P., Amvrosiadis T., Pakan J.M., Rochefort N.L. (2020). Reward association enhances stimulus-specific representations in primary visual cortex. Curr. Biol..

[57] Churchland M.M., Cunningham J.P., Kaufman M.T., Foster J.D., Nuyujukian P., Ryu S.I., Shenoy K.V. (2012). Neural population dynamics during reaching. Nature.

